# Supplementary material for: A Humanized Yeast Phenomic Model of Deoxycytidine Kinase to Predict Genetic Buffering of Nucleoside Analog Cytotoxicity
Source: Genes (Basel). 2019 Sep 30;10(10):770. doi: 10.3390/genes10100770 (PMC6826991; doi:10.3390/genes10100770)
Supplement: Supplementary file 1 [file genes-10-00770-s001.zip › Additional_Files/Additional_File7_GOTermHeatmaps.zip!/Additional_File7_GOTermHeatmaps/single-stranded_telomeric_DNA_binding.pdf]

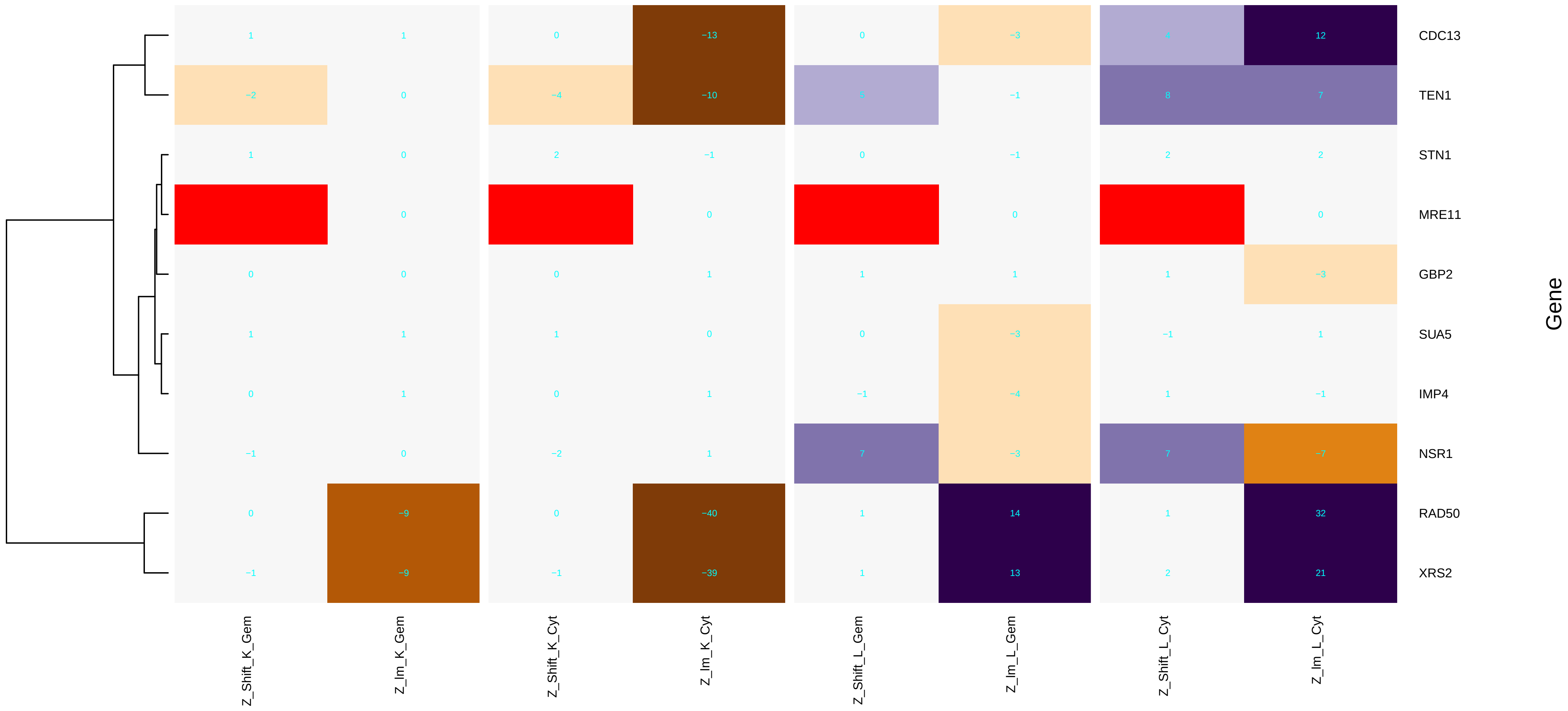
CDC13 1 1 0 −13 0 −3 4 12
TEN1 −2 0 −4 −10 5 −1 8 7
STN1 1 0 2 −1 0 −1 2 2
MRE11 0 0 0 0
GBP2 0 0 0 1 1 1 1 −3
SUA5 1 1 1 0 0 −3 −1 1
IMP4 0 1 0 1 −1 −4 1 −1
NSR1 −1 0 −2 1 7 −3 7 −7
RAD50 0 −9 0 −40 1 14 1 32
XRS2 −1 −9 −1 −39 1 13 2 21
Z_Shift_K_Gem
Z_Im_K_Gem
Z_Shift_K_Cyt
Z_Im_K_Cyt
Z_Shift_L_Gem
Z_Im_L_Gem
Z_Shift_L_Cyt
Z_Im_L_Cyt
Gene
